# A Systematic Review of Non-Traumatic Spinal Cord Injuries in Sub-Saharan Africa and a Proposed Diagnostic Algorithm for Resource-Limited Settings

**DOI:** 10.3389/fneur.2017.00618

**Published:** 2017-12-08

**Authors:** Abdu Kisekka Musubire, David B. Meya, Paul R. Bohjanen, Elly Tebasooke Katabira, Patrice Barasukana, David R. Boulware, Ana-Claire Meyer

**Affiliations:** ^1^College of Health Sciences, Infectious Disease Institute, Makerere University, Mulago Hospital Complex, Kampala, Uganda; ^2^Medicine, Mulago National Referral Hospital, Mulago Hospital Complex, Kampala, Uganda; ^3^Medicine, College of Health Sciences, Makerere University, Mulago Hospital Complex, Kampala, Uganda; ^4^Division of Infectious Diseases and International Medicine, Medicine, University of Minnesota, Minneapolis, MN, United States; ^5^Neurology, University Teaching Hospital of Kamenge, Bujumbura, Burundi; ^6^Neurology, Yale University, New Haven, CT, United States

**Keywords:** non-traumatic, spinal cord injury, myelopathy, sub-Saharan Africa, paraplegia

## Abstract

**Background:**

Non-traumatic myelopathy is common in Africa and there are geographic differences in etiology. Clinical management is challenging due to the broad differential diagnosis and the lack of diagnostics. The objective of this systematic review is to determine the most common etiologies of non-traumatic myelopathy in sub-Saharan Africa to inform a regionally appropriate diagnostic algorithm.

**Methods:**

We conducted a systemic review searching Medline and Embase databases using the following search terms: “Non traumatic spinal cord injury” or “myelopathy” with limitations to epidemiology or etiologies and Sub-Saharan Africa. We described the frequencies of the different etiologies and proposed a diagnostic algorithm based on the most common diagnoses.

**Results:**

We identified 19 studies all performed at tertiary institutions; 15 were retrospective and 13 were published in the era of the HIV epidemic. Compressive bone lesions accounted for more than 48% of the cases; a majority were Pott’s disease and metastatic disease. No diagnosis was identified in up to 30% of cases in most studies; in particular, definitive diagnoses of non-compressive lesions were rare and a majority were clinical diagnoses of transverse myelitis and HIV myelopathy. Age and HIV were major determinants of etiology.

**Conclusion:**

Compressive myelopathies represent a majority of non-traumatic myelopathies in sub-Saharan Africa, and most were due to Pott’s disease. Non-compressive myelopathies have not been well defined and need further research in Africa. We recommend a standardized approach to management of non-traumatic myelopathy focused on identifying treatable conditions with tests widely available in low-resource settings.

## Background

Non-traumatic myelopathy is a challenging condition to manage because of the wide differential diagnosis and substantial regional variation (1, 2). This condition is thought to be more common in Africa than in Europe (3). In Sub-Saharan Africa, non-traumatic myelopathy is associated with a high morbidity (about 50% persistent disability) and mortality (around 10% in admission) and has important economic repercussions for patients and health systems (4–7).

Advanced diagnostics, such as the magnetic resonance imaging (MRI) and several cerebrospinal fluid (CSF) studies, are not readily available in many settings in Sub-Saharan Africa. Thus, standard of care diagnostic approaches for the evaluation of patients presenting with non-traumatic myelopathy developed in resource-rich settings are not applicable to primary care settings in sub-Saharan Africa. Recent reviews of spinal cord injury have focused on descriptive epidemiology and have not been structured so that they can inform a diagnostic approach to non-traumatic myelopathy (1, 2). The objectives of this review were to describe the causes of non-traumatic myelopathy from sub-Saharan Africa and develop an evidence-based diagnostic algorithm for the evaluation of patients that present with non-traumatic myelopathy in resource-limited settings.

## Methods

We conducted a systematic review designed to identify studies describing the epidemiology of non-traumatic spinal cord injury/myelopathy in Sub-Saharan Africa (8). We searched the Medline and Embase database using Ovid to identify peer-reviewed manuscripts describing the epidemiology and etiology of non-traumatic spinal cord injury, myelopathy, paraplegia, and quadriplegia in a general outpatient or inpatient clinical population. Search terms were: 1. paraplegia.mp; 2. quadriplegia.mp; 3. myelopa*.mp; 4. spinal cord.ab; 5. spinal cord.mp; 6. (1 or 2 or 3 or 4 or 5); 7. exp Africa/; 8. (6 and 7); 9. polio.mp; and 10. (8 not 9). Records were then reviewed for inclusion/exclusion based on title, then abstract, and then full-text. Inclusion criteria were as follows: (i) study conducted in Africa, (ii) published before 1 January 2015, (iii) reports more than one etiologic agent, and (iv) described the etiology of non-traumatic myelopathy in a general clinical population by disease process. Exclusion criteria were as follows: (i) study only included children <12 years of age, (ii) could not calculate the frequency of each etiology with data provided in the manuscript, (iii) study included less than 30 individuals, (iv) sample identified from a record review of imaging studies, or (v) studies exclusively focused on radiological or anatomical terminology, and (vi) study conducted outside Sub-Saharan Africa. Two authors (Abdu Kisekka Musubire and Ana-Claire Meyer) independently reviewed the list of titles and abstracts and selected manuscripts for inclusion. Any discrepancies were discussed until consensus was reached. Data on clinical setting, study methods, diagnostic investigations, and frequencies of etiologies were abstracted from the manuscripts (Abdu Kisekka Musubire).

### Statistical Analysis

We described the proportion of etiologies of non-traumatic myelopathy and summary averages were calculated from the weighted mean with 95% confidence interval (95%CI). We excluded polio infection, trauma, somatoform disorders, or diagnosis not localizing to the spinal cord and roots.

Subsequently, a diagnostic algorithm was generated focusing on identifying the most common treatable causes in this region. Other considerations were efficient triage of patients that should be treated by other clinical services such as neurosurgery or orthopedics and the cost and availability of diagnostic tests in typical sub-Saharan African hospitals.

## Results

**Figure 1:**
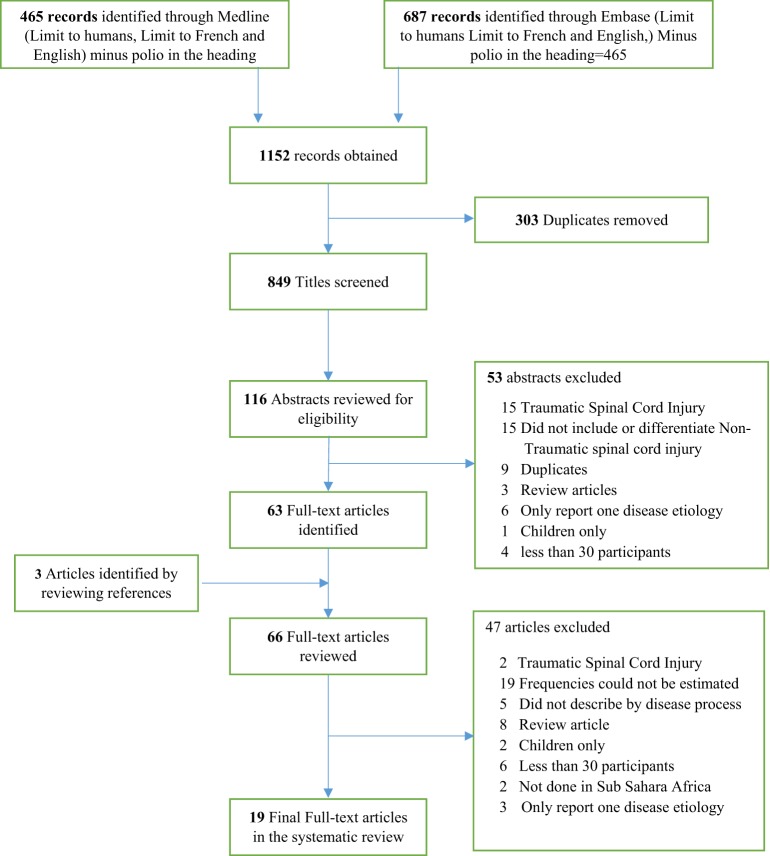
Search strategy in Medline and Embase using OVID search strategy. Flow diagram of the Search strategy through OVID in both the Medline and Embase database going through the selection criteria.

We identified 19 studies (Figure 1) that met our selection criteria. These studies were primarily retrospective, occurred over a large time span (1970–2010) and were conducted predominantly in inpatient tertiary centers in variety of settings including medical, neurological, and neurosurgical wards (Table 1). Only 10 of the 54 countries that comprise the African continent were represented. Most of the studies were done in the West African region (9/19), followed by the East African region (8/19) and only two studies from the South African region. Most of the studies were retrospective (15/19) case series. Only 5 studies specifically focused on myelopathy with the majority covering paraplegia/paraparesis. Several studies (6/19) had less than 100 participants.

**Table 1:** Study methodology: non-traumatic spinal cord injury.

	Article overview						Demographics		Presentation	
Reference	Study period	Country	Study setting	Study design	Study population	*N*	Age	Sex (% male)	Paraplegia (%)	Time (weeks)
Zenebe (4)	1990–1993	Ethiopia	Medical Wards	Retrospective	Myelopathies	130	40 (13–69)	64	77	–
Zenebe et al. (9)	1981–1988	Ethiopia	Internal Medicine Ward	Retrospective	Paraplegia/Paraparesis	164	36 (15–70)	63	100	8
Brown (3)	1972–1975	Malawi	Medical Wards	Prospective	Paraplegia/Paraparesis	102	15^b^	69	100	52^c^
Naus et al. (10)	2000–2002	Malawi	Medical Wards	Prospective	Myelopathy	33	34 (9–67)	73	–	–
Leigh (11)	1975–1976	Tanzania	Medical Wards	Retrospective	Myelopathies	90	36 (3–80)	68	82^a^	–
Scrimgeour (12)	1972–1979	Tanzania	Medical Wards	Retrospective	Paraplegia/Paraparesis	100	31 (2–80)	67	100	10
Parry et al. (5)	1989–1994	Zimbabwe	Rehabilitation Centre	Retrospective	Paraplegia/Paraparesis	159	(–) 6–80	54	100	–
Mahomed and Gelfand (13)	1972–1973	Zimbabwe	General Ward	Retrospective	Paraplegia/Paraparesis	100	30 (4–80)	65	100	–
Looti et al. (14)	1999–2006	Cameroon	Neurology/Neurosurgery	Retrospective	Myelopathies	147	45 (1.6)	59	84	2
Nyame (15)	1991–1994	Ghana	Medical Wards	Prospective	Paraplegia/Paraparesis	64	40 (15–82)	59	88^a^	–
Owolabi et al. (7)	2006–2009	Nigeria	Medical Wards	Retrospective	Paraplegia/Paraparesis	98	40 (15)	72	100	8~
Ogunniyi et al. (16)	1988–1993	Nigeria	Neurology/Neurosurgery	Retrospective	Spinal Cord Diseases	104	45 (16)	77	70	47
Osuntokun (17)	1957–1969	Nigeria	Neurology/Neurosurgery	Retrospective	Neurology patients	9,600	–	–	–	–
Jacquin-Cotton et al. (18)	1960–1969	Senegal	Neuropsychiatric Hospital	Retrospective	Paraplegia	457	–	–	100	–
Ndiaye et al. (19)	1972–1987	Senegal	Neurology/Neurosurgery	Retrospective	Compressive Lesions	253	40–50	^d^	–	–
Kassegne et al. (20)	1998–2007	Togo	Neurology ward	Retrospective	Spinal Cord Compression	39	53 (22–79)	77	–	15
Balogou et al. (21)	1995–1999	Togo	Campus Teaching Hospital	Retrospective	Paraplegia/Paraparesis	243	38 (10–75)	56	–	–
Modi et al. (22)	~2010	South Africa	Neurology/Neurosurgery	Prospective	Myelopathies	100				6^e^
					HIV positive	50	35 (8.6)	50	96	–
					HIV negative	50	53 (15.2)	66	87	–
Bhigjee et al. (23)	~2000	South Africa	Neurology Wards	Prospective	HIV positive with myelopathy	33	39.8	39	–	–

*Countries have been group by geographical region and in alphabetical order, paraplegias are presented as a proportion of paraplegia and quadriplegias, age is presented as mean (SD) or median (range), and time is presented as average time to presentation in weeks*.

*^a^Derived from cases of cervicle spondylosis*.

*^b^Age (73.5% of the participants more than 15 years)*.

*^c^84% of the patients presented within 52 weeks. ~55% of the participants presented within 8 weeks*.

*^d^Study stated male predominant*.

*^e^53% HIV-positive and 68% HIV-negative patients presented in less than 6 weeks*.

Among individuals presenting with non-traumatic myelopathy, there was male preponderance and the average age at presentation increased over time, from an average age in the third decade in the 1970s (11, 12) to the fourth decade in the 1990s and 2000s (7, 16). HIV-infected individuals with non-traumatic myelopathy were younger than HIV-negative patients (22, 23). More than 80% initially presented with paraplegia/paresis (14, 22), and more than 40% presented with a complete transverse myelopathy (involvement of the motor, sensory, and autonomic dysfunction) (3, 22).

There was a striking delay from symptom onset to presentation for clinical care in most studies; for example, only 53–68% of patients in South Africa presented within 6 weeks of onset (22). In Tanzania, the mean delay was 8 weeks (12), while in Nigeria and Ethiopia approximately 50% of the patients presented between 8 and 16 weeks after symptom onset (4, 7, 9).

### Diagnostic Approach

In general, the diagnostic approach within each study was not uniform and there were few diagnostics available. Thus, most diagnoses were presumptive or based on clinical criteria (Table 2). Plain radiographs of the spine were used in nearly all studies and were helpful in diagnosing tuberculous osteomyelitis of the vertebrae, or Pott’s disease, and malignancies that cause compressive bone lesions. In a few recent studies, advanced imaging was used; three studies documented the use of CT and five studies an MRI. However, even when CT or MRI was used in a study, only a small proportion of patients were able to access these modalities. This is likely because most studies were derived from retrospective clinical case series where the costs of advanced imaging are borne by the patient and, thus, only those able to afford the study are able to obtain them.

**Table 2:** Diagnostics utilized systematically in the studies on Non-Traumatic Myelopathy in Africa.

Reference	Country	Spinal X-ray	Myelography	CT/CT myelography	MRI	Tissue histology	CSF microscopy	Full blood count	Serum VDRL	ESR	HIV Ab	Stool microscopy
Zenebe (4)	Ethiopia	X	X	–	–	X	X	–	–	X	X	–
Zenebe et al. (9)	Ethiopia	X	6%	–	–	67%	X	–	–	–	–	–
Brown (3)	Malawi	X	X	–	–	X	X	X	–	X	–	X
Naus et al. (10)	Malawi	–	–	–	–	–	–	–	–	–	–	–
Leigh (11)	Tanzania	X	48%	–	–	21%	X	X	–	–	–	–
Scrimgeour (12)	Tanzania	88%	41%	–	–	Variable	62%	X,	X,	X	–	X
Parry et al. (5)	Zimbabwe	X	X	–	–	X	X	X	–	–	–	–
Mahomed and Gelfand (13)	Zimbabwe	X	X	–	–	X	X	–	–	–	–	–
Looti et al. (14)	Cameroon	X	39%	48%	–	X	X	X	–	–	X	–
Nyame (15)	Ghana	X	X	–	–	67%	X	X	X	X	–	X
Owolabi et al. (7)	Nigeria	X	–	–	26%	–	X	X	X	X	82%	–
Ogunniyi et al. (16)	Nigeria	X	X	6%	4%	Rarely	X	X	–	X	–	–
Osuntokun (17)	Nigeria	–	–	–	–	–	–	–	–	–	–	–
Jacquin-Cotton et al. (18)	Senegal	X	X	–	–	X	X	X	–	–	–	–
Ndiaye et al. (19)	Senegal	X	X	–	–	X	X	X	–	–	–	–
Kassegne et al. (20)	Togo	X	5%	90%	5%	–	–	–	–	–	–	–
Balogou et al. (21)	Togo	X	37%	–	–	–	227	X	–	–	–	–
Modi et al. (22)	South Africa	X	–	–	X	X	X^a^	X	X	X	X	–
Bhigjee et al. (23)	South Africa	–	–	–	X	–	X^a^	X	X	–	X	–

*^a^Only two studies extended CSF analysis for autoimmune and infectious causes (22, 23). Three studies performed CSF bacterial cultures (11, 22, 23). Three studies performed HTLV antibody testing (14, 21, 23). Chest X-rays were routinely done in five studies (4, 9, 12, 15, 22). Serum biochemistry was declared done in five studies (5, 7, 11, 15, 22)*.

*VDRL, Venereal Disease Research Laboratory test; ESR, Erythrocyte Sedimentation Rate; HIV abs, HIV antibody assays; CSF, Cerebral spinal fluid; MRI, magnetic resonance imaging; CT, computed tomography. X means test done in all patients, – means test was not declared done in the methodology. Tissue histology by Biopsy*.

Cerebrospinal fluid microscopy was commonly employed, though only two studies from South Africa were able to perform more comprehensive evaluations for infections, including microbial cultures or polymerase chain reaction (PCR) testing. A few studies did stool and urinalysis in search of schistosomiasis. Several studies from neurosurgical units had histological diagnoses, as they were drawn from samples of surgical cases with biopsies.

### Etiology

Our initial approach to categorizing etiologies of non-traumatic spinal cord injury/myelopathy was drawn from consensus guidelines published by the International Spinal Cord Injury (ISCI) group (24). However, the ISCI approach relies heavily on MRI that was not generally available in the studies we reviewed. Thus, we adapted the ISCI approach for a sub-Saharan African setting as follows. The diagnoses were initially grouped into three broad categories: extramedullary or compressive, intramedullary or non-compressive, and unclear etiology (Table 3). We then categorized extramedullary or compressive lesions into bone or non-bone compressive lesions on the basis of plain radiographs of the spine, myelogram, and the stated diagnosis in the reviewed publication. Finally, we identified etiologies of intramedullary or non-compressive lesions based on laboratory evaluation.

**Table 3:** Common diagnosis according to classification of the causes of Non-traumatic spinal cord injury in Sub-Saharan Africa.

Reference	Country	African region	Number	Myelopathy number (*N*^a^)	Extramedullary myelopathy	Intramedullary myelopathy	Unclear etiology^b^
Zenebe (4)	Ethiopia	Eastern	130	130	76 (58%)	54 (42%)	0 (0%)
Zenebe et al. (9)	Ethiopia	Eastern	164	142	110 (77%)	32 (23%)	0 (0%)
Brown (3)	Malawi	Eastern	102	94	68 (72%)	23 (24%)	3 (3%)
Naus et al. (10)	Malawi	Eastern	33	26	3 (12%)	23 (88%)	0 (0%)
Leigh (11)	Tanzania	Eastern	90	90	48 (53%)	18 (20%)	24 (27%)
Scrimgeour (12)	Tanzania	Eastern	100	96	69 (72%)	15 (16%)	12 (13%)
Parry et al. (5)	Zimbabwe	Eastern	159	148	105 (71%)	31 (21%)	12 (8%)
Mahomed and Gelfand (13)	Zimbabwe	Eastern	100	65	44 (68%)	21 (32%)	0 (0%)
Looti et al. (14)	Cameroon	Western	147	147	98 (67%)	18 (12%)	31 (21%)
Nyame (15)	Ghana	Western	64	55	39 (71%)	11 (20%)	5 (9%)
Owolabi et al. (7)	Nigeria	Western	98	85	52 (61%)	19 (22%)	14 (16%)
Ogunniyi et al. (16)	Nigeria	Western	108	108	73 (68%)	31 (29%)	4 (4%)
Osuntokun (17)	Nigeria	Western	9,600	1,536	750 (49%)	707 (46%)	79 (5%)
Jacquin-Cotton et al. (18)	Senegal	Western	457	312	123 (39%)	48 (15%)	141 (45%)
Ndiaye et al. (19)	Senegal	Western	253	251	238 (95%)	13 (5%)	0 (0%)
Kassegne et al. (20)^c^	Togo	Western	39	39	39 (100%)	N/A	N/A
Balogou et al. (21)	Togo	Western	243	124	92 (75%)	19 (15%)	13 (10%)
Modi et al. (22)	South Africa	Southern	100	96	70 (73%)	26 (27%)	0 (0%)
Bhigjee et al. (23)^c^	South Africa	Southern	33	33	N/A	32 (97%)	1 (3%)
Summary, mean	Africa	All	12,020	3,577	2,097 (58.6%)	1,141 (31.9%)	339 (9.5%)
Estimate 95% CI					57.0–60.2%	30.4–33.5%	8.5–10.5%

*^a^Excludes trauma, functional, “other causes of weakness,” and polio from the denominator for non-traumatic myelopathy etiologies. Polio cases were excluded *n* = 248 (17), *n* = 25 (18), *n* = 5 (3), and *n* = 1 (5). N/A = Two studies had specific enrollment criteria with one only recruiting compressive lesions (20); and the other enrolling only non-compressive lesions (23)*.

*^b^Probable myelopathy based on clinical scenario*.

*^c^Studies included in the weighted means calculation even though they had restricted inclusion criteria. Percentage totals may add up to < or >100% due to rounding off*.

In total, we found over 200 different etiologies described in the included studies (Appendix S1 in Supplementary Material) though this was dependent on the availability of diagnostics. The different etiologies were then grouped in different categories (Appendix S2 in Supplementary Material). Extramedullary lesions constituted a weighted average of 58.6% (range: 12–95%) (Table 4). Compressive bone lesions made up the majority of cases of non-traumatic myelopathy in Africa with a weighted average of 49.1% (range: 12–83%) while non-bone compressive lesions constituted 9.5% (range: 0–16%) of the lesions (Table 4). Nearly 31.9% (range: 5–97%) had non-compressive lesions and no definitive diagnosis was identified in 9.5% (range: 0–45%) (Table 3). A clinical diagnosis of transverse myelopathy unclassified was the most common cause of non-compressive lesions at 4.6% (range:1–23%) (3, 5, 11, 14, 15). Typically, a diagnosis of transverse myelitis was based on lack of a compressive lesion visualized on plain radiograph and inflammatory CSF. Importantly, the underlying causes of transverse myelitis were not identified in most studies.

**Table 4:** Extramedullary causes of non-traumatic spinal cord injury.

Reference	Country	N*	Bone lesions								Non-bone lesions
			TB	Other infections^a^	Primary neoplasm	Metastatic neoplasm	Epidural tumors	Hematologic neoplasm	Degenerative disorders	Intradural (extramedullary)	Congenital anomaly
Zenebe (4)	Ethiopia	130	35 (27%)	–	–	14 (11%)	1 (0.8%)	6 (4.6%)	13 (10%)	7 (5.4%)	–
Zenebe et al. (9)	Ethiopia	142	77 (54%)	2 (1.4%)	1 (0.7%)	11 (7.7%)	–	–	8 (5.6%)	11 (7.7%)	–
Brown (3)	Malawi	94	33 (35%)	5 (5%)	2 (2%)	6 (6%)	1 (1%)	7 (7%)	5 (5%)	8 (8%)	1 (1%)
Naus et al. (10)	Malawi	26	–	–	–	–	–	3 (12%)	–	–	–
Leigh (11)	Tanzania	90	13 (14%)	10 (11%)	–	11 (12%)	–	–	14 (16%)	–	–
Scrimgeour (12)	Tanzania	96	54 (56%)	2 (2%)	2 (2%)	8 (8%)	–	–	2 (2%)	1 (1%)	–
Parry et al. (5)	Zimbabwe	148	43 (29%)	10 (7%)	14 (9%)	13 (9%)	2 (1%)	–	8 (5%)	21 (14%)	2 (1%)
Mahomed and Gelfand (13)	Zimbabwe	65	21 (32%)	1 (1.5%)	1 (1.5%)	9 (14%)	–	3 (4.6%)	1 (1.5%)	5 (7.7%)	3 (4.6%)
Looti et al. (14)	Cameroon	147	19 (13%)	–	5 (3.4%)	25 (17%)	4 (2.7%)	21 (14%)	6 (4.1%)	17 (12%)	–
Nyame (15)	Ghana	55	19 (35%)	–	–	3 (5.5%)	–	6 (11%)	10 (18%)	–	–
Owolabi et al. (7)	Nigeria	85	44 (52%)	–	–	4 (4.7%)	–	2 (2.4%)	2 (2.4%)	–	–
Ogunniyi et al. (16)	Nigeria	108	26 (24%)	–	–	8 (7.4%)	–	6 (5.6%)	31 (29%)	2 (1.9%)	–
Osuntokun (17)	Nigeria	1,536	406 (23%)	6 (0.3%)	–	1 (0.1%)	41 (2.3%)	–	65 (3.6%)	22 (1.2%)	207 (13%)
Jacquin-Cotton et al. (18)	Senegal	312	61 (18%)	16 (4.7%)	5 (1.5%)	24 (7.1%)	–	5 (1.5%)	7 (2.1%)	5 (1.5%)	–
Ndiaye et al. (19)	Senegal	251	N/A	54 (22%)	21 (8.4%)	97 (39%)	37 (15%)	–	–	22 (8.8%)	4 (1.6%)
Kassegne et al. (20)	Togo	39	7 (18%)	–	–	17 (44%)	–	–	15 (38%)	–	–
Balogou et al. (21)	Togo	124	17 (14%)	–	10 (8.1%)	23 (19%)	–	–	42 (34%)	–	–
Modi et al. (22)	South Africa	96	30 (31%)	–	–	17 (18%)	–	6 (6.1%)	15 (16%)	2 (2%)	–
Bhigjee et al. (23)	South Africa	33	N/A	N/A	N/A	N/A	N/A	N/A	N/A	N/A	N/A
Summary		3,577	905 (25%)	106 (3.1%)	61 (1.7%)	291 (8%)	86 (2.4%)	65 (1.8%)	244 (6.8%)	123 (3.4%)	217 (6.1%)

Numbers represent *N* (%). N* Excludes trauma, functional, “other causes of weakness,” and polio from the denominator for non-traumatic myelopathy etiologies.

*^a^Other infections causing bone lesions included: brucellosis, histoplasmosis, and arachnoiditis*.

*Other causes reported included: metabolic bone diseases were reported as etiologies in four persons: *n* = 1 (0.7%) (14), *n* = 1 (0.5%) (19), and *n* = 2 (0.1%) (17). Vascular disorders were reported as etiologies in three persons: *n* = 1 (1.6%) (10) and *n* = 2 (0.8%) (24). Kassegne et al. only included only patients with compressive bone lesions (20) while Bhigjee et al. only included patients without extramedullary lesions (23). Ndiaye et al. only enrolled compressive lesions but excluded Tb spondylitis, hence exaggerating the percentage of neoplasms in this study (19). Naus et al. only included patients without a known cause of myelopathy (10)*.

#### Compressive Lesions

The most common causes of compression were Pott’s disease and tumors (Table 4). Pott’s disease caused 25% of non-traumatic myelopathies (range 13–54%) and 50% of compressive bone lesions. The diagnosis was typically based on finding anterior wedging of the vertebral body on plain radiograph. The clinical presentation of Pott’s disease in these studies differed somewhat from traditional teaching. For example, in a study by Zenebe et al. from Ethiopia, few patients presented with constitutional symptoms such as weight loss and fever that are typically associated with disseminated tuberculosis though 30% of the patients had TB at other sites as well (9). The most common presentation of Pott’s disease in this study was gradual onset of weakness in the legs (92%), vertebral deformity (88%), and vertebral tenderness (88%) (9). Occasionally, individuals reported sudden onset of paraplegia (7.8%) (9).

The weighted average proportion of non-traumatic myelopathies explained by neoplasms that included primary bone neoplasms, metastatic disease, epidural tumors, hematological neoplasms, unclassified intradural, and intramedullary tumors was 18.6% (range 0–75%). The proportion of neoplasms varied depending on the source of the study population. Neoplasms were found at frequencies as high as 49% in a neurology/neurosurgical unit in Cameroon (14), 28% in a neurorehabilitation Centre in Zimbabwe (5) to between 13 and 25% in most studies done on medical/neurological wards (4, 11, 12, 15). HIV prevalence may also contribute to this heterogeneity; in a South African study, HIV-uninfected individuals had a higher proportion of neoplasms than an HIV-infected group (22). Neoplasms were also more frequent in older age groups and presented at a mean age about 50 years (9, 12).

When differentiated from primary tumors, metastatic disease was the second most common cause of compressive bone lesions, representing a weighted average of 8.1% of non-traumatic myelopathies (range 0–44%). Prostatic carcinoma was the most common primary tumor reported in Malawi (3), South Africa (22), and Nigeria (7) while lymphoma was the most common primary tumor in studies from Cameroon and Ghana (14, 15). Hepatocellular carcinoma was predominant in studies from Cameroon (14) and Ethiopia (9). The high proportions of cases of hepatocellular cancer in these regions have been attributed to the high endemicity of hepatitis B (14). Other common primary tumors identified were from the lung and breast (14).

Primary spinal and vertebral tumors were reported less frequently than metastatic disease and ranged from 0 to 20% with a weighted average of 4.6% (9, 11). The most frequently reported tumors were nerve sheath tumors (Schwannoma, neurofibroma, and meningioma) and plasmocytoma (3, 14). Other primary tumors including lipoma, medulloblastoma, and ependymoma were rarely reported (9).

Infections other than tuberculosis that resulted in compressive lesions were less frequently reported. Spinal epidural abscess was only reported (1%) of patients (3). Brucella spondylitis has also been described (9), presenting with a variety of presentations from myelitis to epidural abscesses to spondylitis. Two cases of Cysticercosis and one case of blastomycosis were observed in Zimbabwe (13) and one report of actinomycosis was reported in Tanzania (12).

Degenerative bone disease was infrequently reported and the weighted average was 6.8% (range: 2–38%). Cervical spondylosis occurred more frequently in HIV-negative and older individuals more than 50 years old (14, 22).

#### Non Compressive Lesions

Intramedullary spinal cord lesions, which are non-compressive, constituted 31.9% (range 5–97%) of non-traumatic myelopathies (Table 5). In Western Africa, the majority of intramedullary lesions were suspected to be myelopathy from an as yet undefined nutritional cause. However, in many areas, a clinical diagnosis of transverse myelopathy is the most common cause.

**Table 5:** Intramedullary causes of non-traumatic spinal cord injury.

Reference	Country	N*	Neoplastic intramedullary	Transverse myelopathy unclassified	Infection	Inflammatory/autoimmune	Vascular disorders	Nutrition	Miscellaneous causes
Zenebe (4)	Ethiopia	130	3 (2.3%)	29 (22%)	18 (14%)	–	–	1 (0.8%)	6 (2.3%)
Zenebe et al. (9)	Ethiopia	142	10 (7%)	4 (2.8%)	16 (11%)	–	2 (1.4%)	–	–
Brown (3)	Malawi	94	–	7 (7.1%)	6 (6.4%)	1 (1%)	–	3 (3%)	6 (6.1%)
Naus et al. (10)	Malawi	26	–	–	23 (88%)	–	–	–	–
Leigh (11)	Tanzania	90	4 (4.4%)	6 (6.7%)	4 (4.4%)	–	4 (4.4%)	–	–
Scrimgeour (12)	Tanzania	96	2 (2.1%)	1 (1%)	12 (13%)	–	–	–	–
Parry et al. (5)	Zimbabwe	148	–	17 (12%)	3 (2%)	–	3 (2%)	2 (1.3%)	3 (2%)
Mahomed and Gelfand (13)	Zimbabwe	65	1 (1.5%)	15 (23%)	4 (6.2%)	–	–	–	1 (1.5%)
Looti et al. (14)	Cameroon	147	4 (2.7%)	6 (4.1%)	7 (4.8%)	–	–	–	1 (0.7%)
Nyame (15)	Ghana	55	–	7 (13%)	–	–	–	–	4 (7.3%)
Owolabi et al. (7)	Nigeria	85	–	15 (18%)	–	–	1 (1.2%)	3 (3.5%)	0
Ogunniyi et al. (16)	Nigeria	108	–	9 (8.3%)	3 (2.8%)	–	–	13 (12%)	6 (5.6%)
Osuntokun (17)	Nigeria	1,536	–	22 (1.2%)	Polio only	109 (6.1%)	6 (0.3%)	486 (27%)	84 (4.7%)
Jacquin-Cotton et al. (18)	Senegal	312	3 (0.9%)	–	4 (1.3%)	7 (2.1%)	8 (2.4%)	1 (0.3%)	25 (7.4%)
Ndiaye et al. (19)	Senegal	251	11 (4.4%)	–	–	–	2 (0.8%)	–	–
Kassegne et al. (20)^#^	Togo	39	N/A	N/A	N/A	N/A	N/A	N/A	N/A
Balogou et al. (21)	Togo	124	–	13 (10.5%)	–	2 (1.6%)	–	–	4 (3.2%)
Modi et al. (22)	South Africa	96	1 (1%)	12 (12%)	4 (4%)	7 (7.1%)	1 (1%)	1 (1%)	–
Bhigjee et al. (23)	South Africa	33	–	2 (6.1%)	30 (91%)	–	–	–	–
Summary		3,577	39 (1.1%)	165 (4.6%)	134 (3.7%)	126 (3.5%)	27 (0.8%)	510 (14.3%)	140 (3.9%)

N* Excludes trauma, functional, “other causes of weakness,” and polio from the denominator for non-traumatic myelopathy etiologies.

*^#^Only included compressive lesions. Miscellaneous causes included motor neuron disease and syringomyelia, Nutrition causes include B12 deficiency, Konzo and tropical ataxic neuropathy. Transverse myelopathy unclassified included patients diagnosed with unclassified Transverse myelitis and HIV myelopathy. The two studies from Nigeria skewed the observations toward high burden of nutritional causes (16, 17)*.

Intramedullary infections are presented in Table 6. Human T-Lymphotropic Virus 1 (HTLV-1) myelopathy was the most common infection identified in individuals with non-traumatic myelopathy with the following proportions 9.8% in Ethiopia (9), 4.8% in Cameroon (14), and was lowest at 1% in South Africa (22). In studies from Ethiopia (4, 9), Tanzania (12), and Malawi (10), the diagnosis was based on a characteristic clinical presentation of insidious, slowly, and chronically progressive spastic paraparesis, without remission or acute on chronic episodes, frequently associated with paresthesia and hypoesthesia of the lower limbs, lumbago, and sphincter dysfunctions. Some studies based diagnoses on serology (14, 22). A study from South Africa showed 12 (36%) of the patients had HIV/HTLV-1 coinfection (23).

**Table 6:** Intramedullary infections.

Reference		N*	Total infection	HTLV^a^	Syphilis	VZV	Schistosomiasis	TB
Zenebe (4)	Ethiopia	130	18 (14%)	18	–	–	–	–
Zenebe et al. (9)	Ethiopia	142	16 (11%)	16	–	–	–	–
Brown (3)	Malawi	94	6 (6.4%)	–	6	–	–	–
Naus et al. (10)	Malawi	26	23 (88%)	2	–	–	16	4
Leigh (11)	Tanzania	90	4 (4.4%)	–	–	–	–	4
Scrimgeour (12)	Tanzania	96	12 (13%)	2	4	–	6	–
Parry et al. (5)	Zimbabwe	148	3 (2%)	–	1	–	1	–
Mahomed and Gelfand (13)	Zimbabwe	65	4 (6.2%)	–	2	–	–	–
Looti et al. (14)	Cameroon	147	7 (4.8%)	7	–	–	–	–
Nyame (15)	Ghana	55	–	–	–	–	–	–
Owolabi et al. (7)	Nigeria	85	–	–	–	–	–	–
Ogunniyi et al. (16)	Nigeria	108	3 (2.8%)	–	–	1	–	–
Jacquin-Cotton et al. (18)	Senegal	312	4 (1.3%)	–	4	–	–	–
Ndiaye et al. (19)	Senegal	251	–	–	–	–	–	–
Kassegne et al. (20)	Togo	39	–	–	–	–	–	–
Balogou et al. (21)	Togo	124	–	–	–	–	–	–
Modi et al. (22)	South Africa	96	4 (4%)	1	–	2	–	–
Bhigjee et al. (23)	South Africa	33	30 (91%)	12	2	3	2	6
Summary			134	58 (43%)	19 (14%)	6 (4.4%)	25 (19%)	14 (10%)

N* Excludes trauma, functional, “other causes of weakness,” and polio from the denominator for non-traumatic myelopathy etiologies.

*^a^All cases of tropical spastic paraparesis were categorized under HTLV*.

*This table presents the proportion of patients with intramedullary infections. All cases of tropical spastic paraparesis were categorized under HTLV. The denominator used in this calculation was total number of patients with infections. No intramedullary infectious etiologies were identified in five studies (7, 15, 19–21). Polio cases were excluded *n* = 248 (17), *n* = 25 (18), *n* = 5 (3), and *n* = 1 (5). The following were diagnosed in one patient with a percentage contribution of 0.7%: *Cryptococcus* (10), Cytomegalovirus (CMV) (22), *Klebsiella* (16), *Dracontiasis* (16). The following were diagnosed in two patients with a percentage contribution of 1.4% each: herpes simplex virus (HSV) (23), and cysticercosis (13). The following were diagnosed in three patients with a percentage contribution of 2.2%: Enterovirus (23)*.

Neurosyphilis was identified in three studies: 6% of patients from Malawi (3), 4% from Tanzania (12), and 0.6% from Zimbabwe (5). The diagnosis was typically based on a positive CSF Venereal Disease Research laboratory test (7, 15) but also on MRI showing multiple enhancing granulomata of the cord or as a differential in patients with arachnoiditis (23).

Schistosomiasis due to *Schistosoma mansoni* most commonly affects the conus medullaris and cauda equine (23). There are multiple individual case reports of acute paraparesis in returning travelers from high-income countries where *Schistosoma* ova have refluxed into the vertebral venous system, become lodged, and generated an inflammatory reaction, mimicking transverse myelitis. Yet, the attributable burden of Schistosomiasis toward non-traumatic spinal cord disease in Sub-Saharan Africa is less clear. Studies used widely differing diagnostic criteria ranging from residing in an endemic area with no other obvious cause of myelopathy, microbiological confirmation of systemic disease, *Schistosoma* CSF serology to MRI findings suggestive of schistosomiasis. One study from Tanzania found that 6% of individuals with non-traumatic spinal cord injury had *Schistosoma* eggs in urine, stool, and rectal snips (12). Schistosomiasis was mentioned in one patient in Zimbabwe (5). Nau et al. in Malawi diagnosed 16 out of 33 patients with schistosomiasis based on immunodiagnostic methods in an endemic region (10). It was diagnosed in two patients in South Africa based on MRI findings of the lumbosacral spine showing enhancement of the roots and a swollen conus with hyperintensity on T2-weighted MRI. One had a peripheral eosinophilia and dramatically improved on praziquantel (23).

Two studies from South Africa noted that HIV vacuolar myelopathy was found in 1–6% of patients; diagnosis was based on low CD4+ T-cell count, high HIV viral load, MRI that showed hyper-intense signals on T2 signal intensity in the dorsal and lateral columns, and exclusion of other causes of myelopathy (22, 23). HIV myelopathy has been described in 3–16.9% of patients with myelopathy in Ethiopia and South Africa (9, 23). Other studies that did not have access to advanced imaging defined HIV myelopathy as an HIV positive patient with no other cause of myelopathy identified. In these studies, the proportion of myelopathy patients with HIV infection ranged from 14.1% in Nigeria (7), 30% in Ethiopia (9), to 50% in South Africa (22). This mirrors the prevalence of HIV in the general population.

The other infectious causes that were identified included cytomegalovirus infection (CMV) (one patient) and varicella zoster (two patients) (22). The diagnosis of varicella myelitis was suggested by the presence of a rash. Other infections described included herpes simplex and enterovirus (23). Tuberculosis (six patients) was also described and presented as a non-specific myelopathy with CSF findings suggestive of Tuberculous meningitis or with extensive lymphadenopathy (23).

Autoimmune causes of transverse myelitis were only defined in detail in one study by Modi et al. In a population of 100 patients, 4 HIV-infected patients had acute demyelinating encephalomyelitis diagnosis based on spine and Brain MRI, one had systemic lupus erythematosus, and two HIV-infected patients had presumed neuromyelitis optica based on imaging and CSF findings only. In the same study, four HIV-negative patients were diagnosed with idiopathic transverse myelitis (22).

Vascular, metabolic, nutritional, and other causes for non-traumatic spinal cord injury were relatively rare and no consistent testing was performed in most studies. Spinal cord infarction was confirmed in one patient from South Africa (22), two post-partum mothers in Zimbabwe (5), one in Nigeria (7), one in Ethiopia (9), and in 4% of the patients in Tanzania (11). Metabolic conditions were not reported in most of these studies but potential etiologies include Konzo, neurolathryism, and B12 deficiency. There is literature about Konzo due to cassava consumption in Mozambique, Tanzania, Democratic Republic of the Congo, and Central African Republic (25–30). Konzo is diagnosed by its epidemiologic pattern, nutritional habits in addition to serum and urine thiocyanate concentrations (27). Neurolarythrism due to ingestion of *Lathyrus sativus* plant has been described in Ethiopia (28, 31–33). Vitamin B12 deficiency has been described in a 1–3% of the patients in South Africa, Cameroon, Nigeria, and Malawi (3, 5, 7, 14, 22). There is a reported suspected nutritional causes of myelopathy in large numbers of patients from Nigeria (16, 17).

### Other Conditions

A condition classified as miscellaneous causes like motor neuron diseases and syringomyelia constituted only a weighted average of 3.9% range 0–7.4% of the patients (3, 5, 15).

### Proposed Diagnostic Algorithm for Non-Traumatic Spinal Cord Injury in Resource-Limited Settings

As we reviewed the results of our systematic review in detail, we noted several findings with important implications for a diagnostic algorithm:

1. Age: younger patients are more likely to have an unknown cause or tuberculosis as the cause of their myelopathy while older patients are more likely to have metastatic tumors or degenerative conditions (Figure 3).
2. HIV: HIV-infected patients tend to be younger than HIV-negative patients and are more likely to have infectious etiologies (22, 23).
3. Availability of diagnostics: plain radiographs of the spine, HIV antibody testing, erythrocyte sedimentation rate (ESR), and full hemogram were widely available and generally affordable for patients. Alpha fetal protein and prostatic-specific antigen were also frequently available.
4. Role of ESR: the role of ESR is debated but has been found consistently raised in Potts disease (4, 16).
5. Most common etiologies: Pott’s Disease and metastatic disease are the most common causes of compressive lesions and transverse myelitis of unclear etiology is the most common cause of non-compressive lesions.
6. Cost of diagnostic modalities: the average cost of an MRI in Uganda is 200 USD in a country with a GDP of 714.6 USD and the total expenditure on health as % for GDP (2014) was 7.2%. The health expenditure per capita was 52 USD well below what would be required to investigate such a patient.
7. Availability of treatment: the available treatments include. Anti-tuberculosis medications, Vitamin B12 supplementation, steroids, radiotherapy, anti-cancer drugs, and surgical resection.

**Figure 2:**
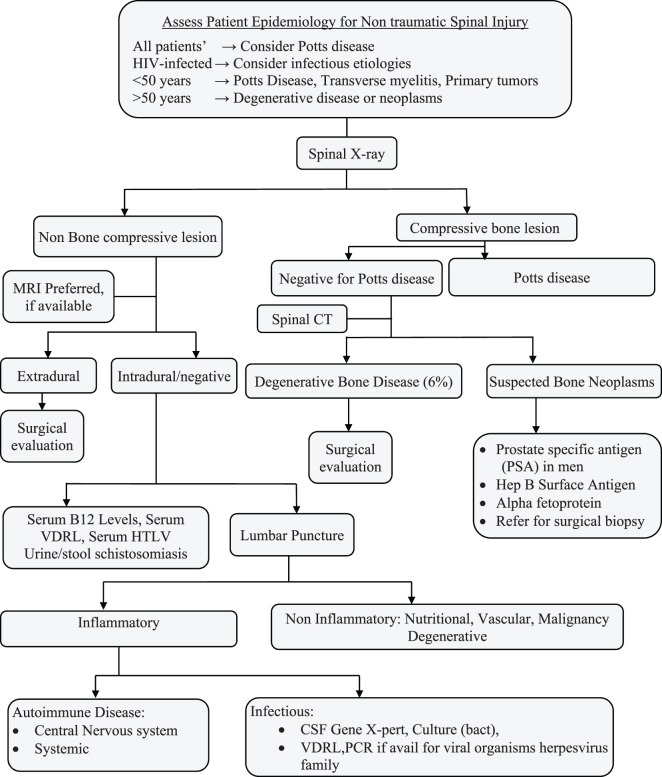
Proposed diagnostic algorithm for non-traumatic spinal cord injury. Proposed diagnostic Algorithm of a patient who presents with non-traumatic myelopathy from the first contact with the health system in the sub-Saharan Africa. CT, computerized tomography; MRI, magnetic resonance imaging; VDRL, Venereal Disease Research Laboratory; PCR, polymerase chain reaction; CSF, cerebral spinal fluid; Hep B, hepatitis B antigen.

**Figure 3:**
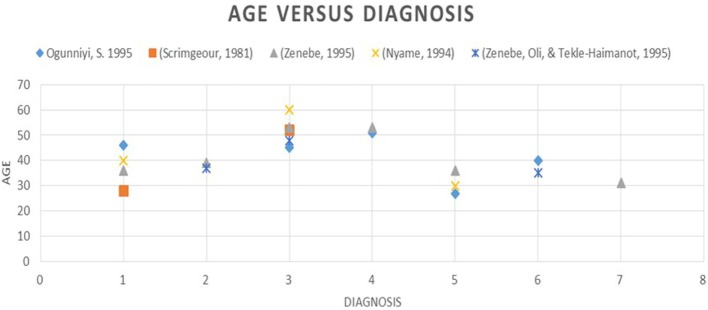
The graph shows age versus diagnosis: 1, TB; 2, primary tumors; 3, metastatic disease; 4, degenerative conditions; 5, TM; 6, HIV myelopathy.

We propose the following algorithm presented in Figure 2.

#### Step 1

##### Screening

###### History and Examination

Spinal cord disorders may present with any combination of the signs and symptoms below:

- Motor deficits: weakness in the limbs, may be symmetric or asymmetric; typically involves both legs, or both legs and both arms together. This is usually accompanied by increased tone, brisk reflexes, clonus, or present Babinski sign (up going toe).
- Sensory deficits: lack of sensation (numbness to touch, temperature, and for advanced practitioners to vibration or position sense) or abnormal sensations (parasthesias/tingling). Like the motor deficits, the sensory deficits may be symmetric or asymmetric; typically involves both legs, or both legs and both arms together. There may be a distinct level on the trunk below which the patient does not have sensation.
- Autonomic nervous system deficits: there may be urinary retention with overflow incontinence, fecal constipation or incontinence, and erectile dysfunction. In very severe cases, there may be paralysis of the muscles of respiration or orthostatic hypotension.

#### Step 2

##### Localize the Lesion

It is of the utmost importance to roughly localize the lesion, so that appropriate imaging studies can be ordered. Because damage to the spinal cord, may be patchy, our clinical examination can only show us the lowest possible spinal level affected. For example, if someone has weakness and sensory loss in both legs, the lesion could be in the lumbar, thoracic, or cervical cord. By contrast, if someone has both arm and leg weakness and sensory loss, the lesion can ONLY be in the cervical cord or above.

1. Lumbar/thoracic/cervical: only legs are affected; may have no sensory level on the trunk.
2. Thoracic/cervical: legs are affected, may have a sensory level on the trunk.
3. Cervical: arms and legs are affected.

#### Step 3

To further evaluate the patient, please refer to Figure 2.

## Discussion

In most of the studies, diagnosis was based on clinical evaluation and on plain radiograph and only in few studies advanced imaging in form of CT/MRI done. However, plain X-rays are limited in the diagnosis of non-compressive myelopathy and other infective etiologies with the exception of Potts disease. Compressive myelopathy of bony lesion was most common etiology and transverse myelopathy of unclassified group were common in non-compressive lesion group.

Pott’s disease is one of the most common causes of non-traumatic myelopathy in sub-Saharan Africa. Tumors, both primary and metastatic, are also common though this may be somewhat biased as several studies were taken from neurosurgical case series (3). Nonetheless, a specific etiology cannot be identified in a large proportion of individuals with non-compressive lesions though most studies had limited investigative capacity. Vascular anomaly of spinal cord cases was missing. In the group of non-compressive lesion, evaluation for vasculitis to be considered. To improve care for individuals with non-traumatic myelopathy in resource-limited settings, there is an urgent need to increase availability of laboratory tests that can identify treatable causes of myelopathy, such as neurosyphilis, HTLV, HSV, VZV, and B12 deficiency. Additional research to better understand the causes of non-traumatic myelopathy in sub-Saharan Africa, especially the treatable causes, is critical to improve care and outcomes from this disorder.

A simple algorithm has the potential to more efficiently triage and evaluate patients in low-resource settings and can assist primary health care providers and non-neurologist physicians in the management of these patients. To improve capacity for the care of patients with non-traumatic myelopathy, it will also be essential to train primary health care providers in neurology, increase post-graduate neurology training opportunities, and increase availability and affordability of MRI and laboratory tests. Raising awareness so that patients present for medical care for non-traumatic myelopathies more quickly will also be important to improve outcomes from this disorder.

## Author Contributions

AKM participated in the study conception, design, data aquisition, data analysis, drafting, revision, final approval, and agreement. DM participated in the study design, data aquisition, revision, final approval, and agreement. PB participated in the study conception, design, revision, final approval, and agreement. EK participated in the study conception, revision, final approval, and agreement. PB participated in the data aquisition, data analysis, drafting, revision, final approval, and agreement. DB participated in the study conception, design, data analysis, drafting, revision, final approval, and agreement. ALM participated in the study design, data aquisition, data analysis, drafting, revision, final approval, and agreement.

## Conflict of Interest Statement

The authors declare that the research was conducted in the absence of any commercial or financial relationships that could be construed as a potential conflict of interest.
