# Supplementary material for: A Systematic Review of Non-Traumatic Spinal Cord Injuries in Sub-Saharan Africa and a Proposed Diagnostic Algorithm for Resource-Limited Settings
Source: Front Neurol. 2017 Dec 8;8:618. doi: 10.3389/fneur.2017.00618 (PMC5770645; doi:10.3389/fneur.2017.00618)
Supplement: Supplementary file 2 [file data_sheet_2.docx]

Appendix B: Mapping diagnoses described in articles to ISCI categories.

| Classification condition | Conditions categorized under the classification |
| --- | --- |
| Arachnoid cysts | Arachnoid cysts of Elsberg, leptomeningeal cyst |
| Spondylitis other | Ankylosing spondylitis, |
| Other infections causing bone lesions | Pyogenic vertebral osteomyelitis and epidural abscess, Actinomycosis and blastomycosis, brucellosis and histoplasma duboissi |
| Liver | Hepatocellular carcinoma, Hepatoma, Liver metastases, |
| Lung | Bronchial Cancer |
| Lipoma | Fibrolipoma, Angiolipoma, Liposarcoma |
| Nerve sheath tumors | Neurofibroma, Neurofibrosarcoma,Shwanoma |
| Metabolic bone disease | Osteodystropihic Fibrosis and Pagets Disease/Platybasia |
| Glioma | Glioblastoma and Glioma |
| Spinal dysraphism | Myelomeningocele, Spinal bifida occulta, Spina bifida manifesta |
| Toxic | Tropical ataxic neuropathy and obscure neuropathy, myelopathy secondary to portal caval shunt |
| Primary vertebral lesions | Aneurysm bone cyst, bone cyst angiosarcoma, angiosarcoma, chondromyoxoma, osteoma, osteoclastoma, sarcoma, reticulum cell sarcoma, ewings sarcoma, hemangioma, Mucopolysaccharidoes type 4 (Morquio's sickness) |
| Other metastatic disease | Metastasis from: anaplastic, melanoma, ovarian, renal, vulva, basal cell, cervix, pancreas, stomach, nasopharyngeal, hematomyeloid |
| Lymphoma | Lymphoma and chloroma |
| Ischemia | Vascular myelopathy, ischemia, intermittent claudication of the cord, aortic aneurysm and vascular disorders unclassified were considered as ischemia |
| Psychomatic | Hysteria, psychomatic,somatoform |
| Vascular | Hemangioma, angioma, hematoma,heamatomyelia |
| Congenital degenerative | spino cerebellar, congenital degenerative |
| Vascular malformations | Vascular malformations includes av malformations |
| HIV myelopathy | Seroconversion was considered as HIV myelopathy as well as vacoular myelopathy |
| Motor Neuron diseases | degenerative myelopathy and motor neuron disease |
| Myopathy | Abnormal potassium and myopathy |
| Transverse myelitis un classified | Transverse myelitis unclassified Viral myelitis was considered under TM un classified |
| Intramedullary Tuberculosis | Other forms of Tb like TBM were considered under intramedullary Tb |
| No diagnosis | Radiculo-myelopathy with a normal myelogram |
|  | Schistosomiasis was included in the intramedullary section |
| Tuberculous spondylitis | All Tb except where indicated was treated as TB spondylitis |
| Vertebral column degenerative disorders | herniated disc, osteoarthritis, Disc disease, spinal canal stenosis, ligamentum flavum hypertrophy, spondylosis,fluorosis, kyphoscoliosis  Disc prolapse, herniated disc,Thickened ligamentum flavum, fluorosis spinal stenosis, fibrous cord compression under spinal cord stenosis  disc degeneration under disc disease,Cervical spondylosis was included in spondylotic myelopathy |
